# Utilization of Vegetable and Fruit By-products as Functional Ingredient and Food

**DOI:** 10.3389/fnut.2021.661693

**Published:** 2021-06-15

**Authors:** Ke Qi Lau, Mohd Redzwan Sabran, Siti Raihanah Shafie

**Affiliations:** Department of Nutrition, Faculty of Medicine and Health Sciences, Universiti Putra Malaysia, Serdang, Malaysia

**Keywords:** vegetable by-products, fruit by-products, food waste recovery, utilization of food waste, functional food, health benefit, food security

## Abstract

With the constant growth of the human population, the global demand for food is increasing annually. Food security is an arising issue due to decreased resources and massive waste production from the agricultural sector. For example, not all parts of fruits and vegetables are consumed by consumers, and this phenomenon can lead to huge amounts of food wastes that are produced globally. Moreover, non-utilized agriculture by-products, including seed coat, hull, husk, peels, seeds, and pomace, can cause environmental issues. Hence, efficiently utilizing food wastes, such as vegetable and fruit by-products, could be a way to increase food sustainability, and in line with the United Nations Sustainable Development Goal (SDG) to ensure sustainable consumption and production patterns. Moreover, certain agriculture by-products are reported to have a high nutritional value and could be potentially used as functional ingredient and food in the food industry. This review article summarizes findings on the development of new functional foods by utilizing different types of agriculture by-products, that is, vegetable and fruit by-products as ingredients. Furthermore, the nutritional values, processing methods, product acceptability, and potential uses of these vegetable and fruit by-products are also discussed. These by-products can be an alternative source of nutrients to support the global demand for functional foods and as one of the strategies to cope with food insecurity. Studies have shown that different types of fruit and vegetable by-products were well-incorporated in the development of functional foods, such as bakery products and dairy products. Of great importance, this review article provides an insight of the nutritional value, health benefits, and utilization of fruit and vegetable by-products.

## Introduction

In the agriculture field, postharvesting, processing, distribution, and consumption sectors could generate agriculture by-products that are wasted in huge amounts, which can contribute to the food waste problem (1). These food wastes are part of unintentional and intentional losses, which can lead to wastage of agriculture by-products (2). The unintentional losses include inadequate farming technologies and lack of proper transportation, whereas the intentional losses include due to human eating habits. Approximately 45% of fruits and vegetables are wasted worldwide, which is one of the categories with the highest wastage rate (1). The highest agricultural wastage is found in Europe, Latin America, North America, and Oceania, and it accounted for ~10% higher than that of industrialized Asia (1). Moreover, the highest production wastage is found in Sub-Saharan Africa, which constitutes ~20% (1).

It is of great concern that a massive amount of agricultural by-products contributes to the food waste problem, subsequently, it can further lead to many environmental and economic issues (1). According to FAO, food loss and waste is the second highest cause of greenhouse gas emission. Statistically, 1.3 billion tons of wasted foods caused the emission of around 4.4 gigatons of greenhouse gas (1). Greenhouse gases are mostly originated from landfill emissions of decaying food, on-farm agriculture emission, electricity and heat in the manufacturing process, and the energy used for agriculture by-products that are lost or wasted. Clearly, these events can cause the greenhouse effect and lead to global warming and climate change, which might further affect the extinction of some animal species (3).

From the economic perspective, agricultural by-products and wastages can affect both the income of farmers and the expenses of consumers. For example, the cost of food waste is very high, especially in countries with a high population such as in China and the United States of America (4). It is evident as foods worth $32 billion are thrown away yearly in China. Moreover, on average $1,600 worth of food are wasted annually from a family of four in the United States of America. These large amounts of food wastes have a big impact on the income of farmers (1). In the sub-Saharan Africa, farmers earn only <$2 in a day due to a large amount of postharvest losses. In fact, $940 billion worth of foods are estimated to be lost or wasted annually, and the World Bank estimated $40 million of economic gains could be achieved with just 1% reduction in postharvest losses, where this can benefit the small farmers (3). The extremely huge amount of wastage has drawn the attention of researchers to look for an alternative of utilizing fruit and vegetable by-products. Therefore, utilizing agricultural by-products not only helps the farmers and economics of customers, but also it can increase food sustainability and reduce food insecurity, especially for the underdeveloped countries. This review summarizes different types of food developed using fruit and vegetable by-products. Through this review article, the potential use of agricultural by-products is explored and discussed.

## Agricultural By-products

Agricultural by-products or wastes are the residues from the growing and processing of raw agricultural products, such as fruits, vegetables, meat, poultry, dairy products, and crops (5). Moreover, the wastes include animal wastes, such as manure and animal carcasses, food processing waste, crop waste, and dangerous and toxic agricultural waste, such as pesticides, insecticides, and herbicides. These agricultural wastes can exist in the form of liquid, slurry, or solid.

It is estimated that about 998 million tons of agricultural wastes are produced annually due to the expanding agricultural production. With the drastic demand, there is a significant increase in livestock waste, agricultural crop residues, and agro-industrial wastes. Hence, the expansion of the farming systems in developing countries increases the global agricultural wastes. It is evident as organic wastes generate up to 80% of the total solid wastes in any form, whereas the manure production can reach up to 5.27 kg/day/1,000 kg live weight, on a wet weight basis (5). Agriculture by-products are also largely contributed by the improper utilization of fruits and vegetables that are commonly overproduced seasonally (6). Vegetable and fruit by-products represent 44% of global food wastes by commodity, whereas roots and tubers contribute that of 20%, and cereal constitutes that of 19% (1, 7). In this review article, two types of agricultural by-products, that is, vegetable and fruit by-products are further discussed on their usefulness as functional ingredient and/or foods.

### Vegetable By-products

Vegetable by-products are the secondary products that are often discarded or wasted during manufacturing or other stages of food processing. Up to one-third of vegetables could be wasted in the preparation process. Interestingly, certain parts of the vegetables are knowingly wasted due to their unfavorable taste or texture. For example, vegetable parts, such as hulls, bagasse, and seeds, are mostly discarded in the production line. For certain types of vegetables, such as broccoli, cauliflower, and pumpkin, the stem and leaves are not consumed and thrown away.

The seed coat, which is the outer layer that covers the seeds or beans, functions to protect them from external damages (8) and is one of the vegetable by-products. Seed coats are usually not consumed due to the texture and taste, except for some thin seed coats, such as peanut seed coats. In the canned food industry, seed coats are discarded in a large quantity as ~22% of oilseeds and pulses worldwide are lost or wasted annually (1). The wastages of oilseeds and pulses are highest in the North Africa, West Asia, and Central Asia regions, and these by-products are mostly lost during the agriculture stage (1).

Unlike the seed coat, the hull is the hard-protecting cover of the seeds or grains that protects them during the growing period. Due to their hardness of texture, hulls are removed before cooking or manufacturing. Globally, hulls contribute to a large amount of food waste, especially rice hulls due to the high consumption of rice among the Asian countries (9). It is reported that 30% of the whole cereals are lost, and most of the wastages are due to human consumption and postharvest activities (1). To overcome this problem, hulls are used as a building material, fuel, and fertilizer (10) as one of the strategies to reduce wastage. Due to its high content of fiber and protein (11–13), the hull can be an alternative source of functional ingredient.

Not all vegetables have peels, unlike fruits. Tubers, gourd, and allium families contain peels, yet they are not normally consumed or used in food preparation. Therefore, vegetable peels are often discarded as kitchen or production wastes in the manufacturing line. Annually, large amounts of tuber and roots are wasted, and up to 5,814,000 tons of tubers and roots are wasted during the consumption stage in the North America and Oceania regions. In addition, Europe, North America, Oceania and industrialized Asia have the highest wastage of roots and tubers at the agriculture stage, where ~45% of tubers and roots are lost globally (1).

### Fruit By-products

Fruit by-product is one of the principal roots of municipal solid waste, which has been the unresolved environmental menace (14). Municipal solid waste is defined as any substances discarded from residential, commercial, and industrial activity (15). Fruit peels or rinds are one of the by-products, which are commonly discarded during the consumption or manufacturing stage. It is the outer layer of fruits that functions to protect the fruits from environmental factors. Some fruits have thick and rough skin, such as pomegranate, whereas some fruits have a thin peel, such as mango. Most of the fruit skins are not consumed by consumers due to the rough texture and bitter aftertaste.

Other fruit by-products, such as seeds, are mostly consumed together with the fruits, such as watermelon and kiwi. In contrast, larger size seeds are not consumed, and they are mostly discarded after the flesh is consumed. Fruit seeds are rich in nutritional sources and have been used to develop functional foods (16). Fruit pomace is the remaining solid matter after juice extraction of fruits and might include seeds, skin, pulps, and stems for certain fruits. For example, apple pomace accounts ~25% of apples, where wastes from the apple juice and cider industry can generate a massive amount of pomace (17). The wastage from apple pomace is especially higher in the Western countries, such as in Germany and New Zealand (18).

## Nutritional Value of Vegetable and Fruit By-products

Findings in the literature showed that vegetable and fruit by-products have high nutritional value (19, 20). Seed coats were reported to contain high dietary fiber. For instance, legumes seed coats are high in dietary fiber, ranging from 65 to 86% (19). Moreover, high content of dietary fiber has been reported in lemon, orange, *bambangan*, and grapefruit peels are also a rich source of dietary fiber (21, 22).

Apple pomace contains high dietary fiber compared to other fruit by-products, such as citrus peel (22). There are studies in the literature showed that apple pomace has been used as a functional ingredient and source of additional fiber in developing biscuits, cakes, and bread (23–26). Furthermore, pectin extracted from apple pomace has been used as an alternative source of gelatinizing agent that is used mainly to produce jam or jelly (27). Tomato pomace, which is high in dietary fiber and minerals, has been used as a functional ingredient to develop several functional foods, such as bread, muffin, and low-calories jam (28) Fuentes et al. (29) showed that the jam added with tomato pomace had 15–20% higher dietary fiber as compared to the commercial apricot jam. Other pomaces, such as grape, contain high protein and dietary fiber (30) and also a rich source of unsaturated fatty acids (31, 32).

Apricot kernel is also a potential functional food ingredient with high nutritional value. Studies in the literature have shown that apricot kernel has a high content of protein. Moreover, the apricot kernel has been used as a fat replacer in cookies and is incorporated into the development of noodles, bread, and cookies as reported in several studies (33, 34).

Moreover, the seed of fruits has been studied extensively. For example, fat extracted from *rambutan* seed showed high thermal stability, which suggested its possible uses in the food industry. The fatty acids of *rambutan* seed fats are mainly composed of oleic and arachidonic acid, which are omega-9 and omega-6 fatty acid, respectively (35). Furthermore, *rambutan* seed fats had a similar composition of fatty acids to cocoa fats and can be used to replace cocoa butter in chocolate production (36).

## Health Benefits of Vegetable and Fruit By-products

### Antioxidant Activity

A study showed that seed coats from nuts and beans contain a high level of phenolic compound, which can contribute to strong antioxidant activity (20). The removal of seed coats of nuts has been associated with the reduction of antioxidant activity up to 90% (20). Further study showed that flavonoids and saponins extracted from the black bean seed coats are well-retained, even after the baking process (37, 38), and these flavanoids and saponins from the black bean seed coat are suitable for food development due to the thermostability properties (39).

Moreover, several types of vegetable peels have shown to exhibit strong antioxidant properties. For example, potato peels were fortified into vegetable oil as antioxidant source in a study by Mohdaly et al. (40). The addition of potato peels improved the hydrolytic stability of vegetable oil and slowed thermal deterioration and stabilized the vegetable oil. In addition, the addition up to 200 ppm of potato peels extract had comparable stabilization efficiency with the synthetic antioxidants. Furthermore, potato peels have been used as a functional ingredient in making cookies, wheat bread, and bakery goods in order to improve the nutritional properties and health benefits of the products (41–44).

Many types of fruit peels and pomace contain high phenolic compounds, which possessed strong radical scavenging activity. Some fruit peels, such as *rambutan*, mango, *bambangan*, and mangosteen peels, are reported to have high antioxidant activity and suitable to be used as natural antioxidants in the development of functional food (21, 45, 46). Apple pomace is a good source of polyphenols and exhibits strong antioxidant activity (47–51). Polyphenols from apple pomace showed stronger radical scavenging activity than vitamin C and E, which indicated that apple pomace could be a potential natural source of antioxidants (49). Apart from that, tomato pomace is a rich source of phenolic compounds and tocopherols (31, 32) for strong antioxidant activity (50, 51). Moreover, grape pomace has been used as a functional ingredient in many types of food development including breads, yogurt, cheese, muffins, salad dressing, cookies, and brownies (52–57). For example, the fortification of grape pomace has enhanced the health benefits of the products, especially in terms of antioxidant activity and increased the nutritional value of products.

### Antimicrobial and Antifungal Activity

There are studies in the literature that found several types of vegetable peels with high antimicrobial and antifungal activities. In the study of Rakholiya et al. (58), phytochemicals of vegetable peels were analyzed, and the peels of *Moringa oleifera* showed the highest antimicrobial activity toward Gram-positive bacteria among all the fruits and vegetable peels studied. Furthermore, compatible results were shown in a study by Chanda et al. (59) in terms of the antimicrobial activity of fruits and vegetable peels. Moreover, lemon, papaya, and sapodilla peels showed high antimicrobial activity, especially toward Gram-negative bacteria, whereas papaya peels showed high inhibition toward fungi (58). Other peels, such as pomegranate peels, contain high antioxidant activity and antimicrobial activity against food-borne pathogens, such as *Listeria monocytogenes, Escherichia coli, Yersinia enterocolitica*, and *Staphylococcus aureus* (46, 60, 61).

Besides its high antioxidant profile, mangosteen seed has antibacterial properties, where its antibacterial activity was highest among all the mangosteen parts (62). Indeed, the mangosteen seed also showed a better inhibition toward Gram-positive bacteria (62).

### Anticancer Activity

Citrus peels with high flavonoids levels possessed anticancer activity. A study lead by Lai et al. (63) showed that citrus peels have protective effects against cancer. Citrus peels significantly reduced the size of tumors of prostate cancer and showed strong anti-inflammatory, antiproliferative, and antiangiogenic activities, and enhanced apoptosis-inducing effects on prostate cancer. The high level of polymethoxyflavones in citrus peel could also effectively suppress azoxymethane-induced colonic tumorigenesis.

### Antidiabetic and Antihypocholesterolemic Activity

Some fruits and vegetables by-products are found to have antidiabetic properties. The antidiabetic effects of mango by-products were investigated in a study by Sarahí et al. (64). Mango by-products from the juice manufacturing industry including mango peels and pulps have shown insulin mimetic effect mainly due to the high level of soluble fiber, polyphenols, and carotenoids. Moreover, onion by-products with high dietary fiber showed hypoglycemic activities by effectively decreasing the starch digestibility and inhibiting the activity of alpha-amylase (65).

In a study by Mildner-Szkudlar and Bajerska (66), bread fortified with grape by-product has shown a positive effect in reducing cholesterol levels in an animal model, where further study is warranted to evaluate its use among humans. In addition, extracts of flavonoid and saponins from black bean seed coats could inhibit cholesterol micellization up to 55.4 ± 1.9%, where the effect was superior compared to stigmasterol (39). Of great interest, the authors added black bean seed coats that can be incorporated to develop cholesterol-lowering functional food and to explore more possibilities of utilization of black bean and other seed coats (39).

Many types of fruit and vegetable by-products have shown to be rich bioactive compounds, which are closely related to the health benefits, such as antioxidant and antimicrobial activity. More future studies could be done to incorporate the vegetable and fruit by-products for the development of new functional food to increase the nutritional security of food in the future market.

## Functional Foods

As people are getting more health conscience, the demand for functional food has increased over the past years. Functional food is defined as “food that has a positive impact on an individual's health, physical performance, or state of mind, in addition to its nutritious value” (67). The American Dietetic Association has defined functional food as “whole, fortified, enriched, or enhanced” food which is consumed as “… part of a varied diet on a regular basis, at effective levels” (68).

In the current market, the Asian Pacific and North American have the largest functional food market, which contributed to 34 and 25%, respectively (69). The United States of America, Europe, and Japan are the top three countries, which contributed to ~82% of the total sales for functional foods and supplements in the year 2003 (70). Functional food started with the fortification of certain vitamins or minerals into the food developments. In recent years, the development of functional foods has further proceeded to the fortification of one or more compounds, which could provide multiple health benefits (71).

## Vegetable and Fruit By-product as Functional Ingredients

### Bread

Bread is a common confectionery product, which is widely consumed worldwide. Based on a published report (72), $358 billion worth of breads were consumed in the year 2016, a constantly increasing trend since 2007. Although breads are originated from Egypt, they are also consumed in Middle Eastern countries. Globally, the United States of America, China, and Russia are the top three countries with the highest bread consumption. These countries accounted for 32.7 million tons of bread consumption, equivalent to ~25% of the total consumption of bread globally in the year 2016. Due to the high consumption of bread worldwide, many studies have incorporated vegetable and fruit by-products to develop functional bread, and these findings are summarized in Table 1.

**Table 1:** Study and main findings of vegetable and fruits by-products utilization as functional ingredients in development of bread.

**Product developed**	**Vegetable and fruit by-products**	**Study and main findings**	**References**
Whole wheat bread	Black bean seed coat (BBSC)	In the study, 0.5% of black bean seed coat (BBSC) was incorporated for the development of whole wheat bread. The study found no significant difference in terms of baking properties and texture parameters after the incorporation of BBSC. However, the crumb color showed significantly lower value in L*, a*, and b*score. Besides, all attributes during the sensory evaluation did not have significant difference with control except color, where the new product obtained higher score than control. The study also showed that more than 80% of flavonoids, saponins and anthocyanins were retained in baked bread, incorporated with BBSC.	(38)
Wheat bread	Flaxseed hulls (FH)	In the study, 1–5% flaxseed hulls (FH) were incorporated into wheat bread. The phenolics content was increased by 93% and the radicals scavenging ability as well as reducing power, increased by 176 and 220%, respectively with the incorporation of 5% of FH. Besides, the proteins digestibility decreased to 8%. Based on the *in vitro* starch digestibility, expected glycemic index was not significantly affected with enrichment of FH. It was found that FH enrichment decreased loaf volume and increased crumb hardness which affected texture of bread. In the sensory evaluation, no significant difference was observed on the overall acceptability of bread with the incorporation of FH up to 3%.	(12)
Bread	Pea hulls (PH)	Up to 10% of small-grained pea hulls (PHS) and coarse-grained hulls (PHC) were incorporated in bread. The study showed that pea hulls (PH) contained high total dietary fiber (TDF), that is, 70.69% in PHS and 93.29% in PHC, respectively. The total baking lost and crumb porosity decreased with increasing amount of both PHS and PHC. The authors found that the functional properties of crust and crumb were affected with the addition of PH. In addition, TDF of bread increased to 17.7 and 21% with the addition of 10% PHS and 10% PHC respectively. In the sensory evaluation, the acceptability of bread decreased with increasing amount of both PHS and PHC. The study found that up to 10% PH could be incorporated in bread, with acceptable physical properties and sensory values, without altering the color.	(73)
Wheat bread	Potato peels (PP)	The authors used 0, 4, 8, and 12% of potato peels (PP) to be incorporated in wheat bread. An increase of PP in the formulation decreased the baking loss and bread volume. The incorporation of 12% PP significantly increased the dietary fiber (5.3–10.8 g/100 g DM) and cellulose (0.5–2.5 g/100 g DM). The study found that the texture profile of bread was affected with the incorporation of PP, where the hardiness, gumminess, modulus and adhesiveness were increased and the deformation energy, and springiness was reduced. Based on the sensory evaluation, 8% PP substitution had a better acceptability.	(44)
Wheat bread	Pomegranate peel (PGP)	Up to 10% of pomegranate peel (PGP) was incorporated in wheat bread. The antioxidant levels of bread increased from 1.8 mmol TEAC per g (0% PGP) to 6.8 mmol TEAC per g (10% PGP) and remained constant under 5 days storage. Besides, the concentration of free radicals decreased with increasing PGP substitution. In the sensory evaluation, no significant difference was observed as compared to the control in terms of acceptability for all attributes at all substitution level except taste and mouthfell of 10% substitution as it caused a significantly lower score. The authors indicated that 2.5% of PGP substitution showed the highest acceptability with acceptable antioxidant value.	(74)
Bread	Tomato pomace (TP)	In this study, 6 and 10 % of tomato pomace (TP) were incorporated in bread. Based on the preliminary analysis, TP contained 174.12 mg/kg lycopene, 32.66 mg/kg β-carotene, 111.89 mg/kg ascorbic acid and 865.77 mg GAE/kg total phenolics. Besides, TP was found to be rich in minerals including potassium, magnesium, calcium and sodium. The moisture content, titratable acidity and bread crumb elasticity were significantly increased with the supplementation of TP, while specific volume and bread crumb porosity were found to be decreased. In term of sensory evaluation, the acceptability of bread was slightly decreased with the increasing supplementation of TP. It was found that bread with 6% TP supplementation showed high sensory results and acceptable physiochemical properties.	(75)
Bread	Grape pomace (GP)	In the study, 2, 5, and 10 of grape pomace (GP) were incorporated in bread. The incorporation of GP significantly increased the antioxidant activity of bread samples, from 11.06% (0% GP) to 55.64% (10% GP). Besides, the total phenolic content of bread increased by more than 150% with the 10% of GP fortification. The study also found that fortification of GP significantly affected the color of crust and crumb and texture of bread. The hardness was increased while the springiness and cohesiveness were decreased with the increasing amount of GP being fortified. In term of sensory evaluation, no significant difference was observed in shape symmetry, crumb color, pore structure and odor for all samples. It was found that 2 and 5% GP-fortified bread had high acceptability, compared to the 10% of GP-incorporated bread.	(54)

L*, L* standard (indicates lightness); a*, a* standard (indicates red/green coordinate); b*, b* standard (indicates yellow/blue coordinate).

It is found that the incorporation of vegetable and fruit by-products increased the nutritional values of the bread. However, the physical and organoleptic acceptability are slightly affected with such incorporation in most of the studies. The color of bread and the texture profile are affected as the bread turns darker or harder, which subsequently affects the overall acceptability of consumers.

### Cookies and Biscuits

Biscuits and cookies are popular confectionery products and are commonly consumed as a sweet dessert instead of savory food. Due to the low water activity and long shelf life, biscuits and cookies are also used as emergency food. However, biscuits and cookies are mostly low in nutritional values, such as fiber, protein, and minerals (76). The incorporation of vegetable and fruit by-products increased the nutritional values of the product (Table 2).

**Table 2:** Study and main findings of vegetable and fruit by-products utilization as functional ingredients in development of cookies and biscuits.

**Product developed**	**Vegetable and fruit by-products**	**Study and main findings**	**References**
*Acha*-based biscuits	Soybean hulls (SH)	In the study, 0, 2.5, 5.0, and 7.5% of soybean hulls flour (SH) was incorporated into *acha*-based biscuits. The incorporation of SH significantly affected the physical and functional properties of biscuits. It is found that an increment in SH level (0–7.5%) increased the water absorption (1.50–1.92 g/g) and the emulsifying capacities (50.27–53.41%), while decreased the bulk density (0.68–0.65 g/ml) and the oil absorption capacity (1.49–1.46 g/g). Besides, the incorporation of SH significantly increased the level of fiber (0.71 to 0.91%) and protein content (3.91 to 5.39%) while decreased the carbohydrate content (69.07 to 59.66 %) of the *acha*-based biscuit. In sensory evaluation, there was no significant difference in term of color, crispiness, aroma and general acceptability after the incorporation of SH. Nevertheless, 7.5% fortified biscuits had the highest acceptability in term of texture, which was significantly higher compared to the control. Nevertheless, the authors showed that 2.5% fortified biscuits were most preferred organoleptically.	(11)
Biscuits	Potato peels fiber (PF)	In the study, 0, 5, 10, and 15% of potato peel fiber (PF) was incorporated in biscuits. Preliminary analysis showed that the dried potato peel contained 76.40% dietary fiber and 14.04% protein. The incorporation of PF increased the carbohydrate, ash and fat content while reduced the protein content of biscuits. Based on the sensory evaluation, acceptability of biscuits decreased with the increasing PF content. The authors found that 5% formulation was preferable as more than 5% incorporation resulted in dark crumb color, hard texture which subsequently affected the appearance and mouthfeel.	(42)
Cookies	Grape pomace (GP)	In the study, 20 to 30% of grape pomace (GP) was incorporated in cookies formula. The fortification significantly affected the color and texture of cookies. It was found that the dietary fiber, total phenolic content (TPC) and free-radical scavenging activity of cookies increased with the increasing fortification of GP. The dietary fiber of fortified products ranged from 3.17 to 5.82 g/100 g and TPC of cookies increased from 23.98 mg GAE/100 g (20% GP) to 48.08 mg GAE/100 g (30% GP). Based on the sensory evaluation, the fortification of GP did not significantly affect the overall acceptability of cookie samples.	(55)
Cookies	Grape pomace (GP)	In this study, 5, 10, 15, and 20% of GP were incorporated in cookies. It was found that anthocyanin, total phenolic content (TPC), flavonoid, tannins and antioxidant activity of cookies significantly increased with increasing GP content. In addition, the color intensity was increased with the increasing GP amount and the maximum color intensity was found in cookies with 20% GP, where the fortification produced brown color in cookies. In term of sensory evaluation, 5% and 10% GP fortified cookies had significantly higher overall acceptability than control. Besides, 5% GP fortified cookies had the highest organoleptic score in all attributes. The study showed that 20% GP-fortified cookies with highest color intensity had the highest acceptability for color compared to others.	(56)
Cookies	Pomegranate peel (PGP)	Up to 7.5% of pomegranate peel (PGP) was incorporated in cookies and the dietary fiber increased from 0.32 to 1.96 g/100 g with addition of 7.5% PGP, which is more than 6 times higher than control. The supplementation of PGP significantly increased the minerals content, that is, calcium, sodium, potassium, iron, and zinc in cookies. It was found that the total phenolic compounds and antioxidant activity of cookies significantly increased with the increasing amount of PGP. Besides, the addition of PGP reduced the level of oxidative degradation where cookies with 7.5% PGP showed a lower free fatty acid (0.20%) after 4 months of storage, compared to control samples (0.22%) at the 2nd month of storage. Although the overall acceptability of cookies of PGP was lower than control, it remained acceptable under supplementation of 7.5%.	(76)
Biscuits	Pomegranate peel (PGP)	Up to 10% of pomegranate peel (PGP) was incorporated in the biscuits. It was found that PGP had considerable amount of dietary fiber, total polyphenol, β-carotene, calcium and iron. An increase of PGP incorporation significantly increased the hardness and decreased the cohesiveness and springiness of biscuits dough. The incorporation of PGP affected functional properties of biscuits where breaking strength was found to be increased, with a decreased spread ratio. Based on the sensory evaluation, biscuits with 7.5% PGP addition were well-accepted.	(77)
Cookies	Apricot kernel (AK)	In the study, up to 25% of apricot kernel (AK) were incorporated in the development of low-fat cookies as a fat replacer. The incorporation of AK significantly increased the total dietary fiber content of cookies and altered the color and texture of cookies. The hardness of cookies increased with increasing AK amount being incorporated, while the spread ratio was found to be decreased from 7.10 (0%) to 5.89 (25%). In the sensory evaluation, no significant difference was observed for overall sensory score at all concentrations of AK substitution.	(78)

As can be seen in Table 2, cookies and biscuits that were incorporated with vegetable and fruit by-products had improved nutritional properties, such as dietary fiber and minerals. However, high-level incorporation could affect the texture and sensory results of cookies and biscuits. Based on the findings (Table 2), the incorporations of vegetable and fruit by-products up to 10% produced high acceptability and high functional values including emulsifying capacities and bulk density. Despite the slight changes in the color and texture, a low-level incorporation of vegetable and fruit by-products into the development of cookies and biscuits could be a good alternative for healthy snacks.

### Noodles

Noodles have a history of more than 4,000 years, and it is one of the most popular staple foods, especially in the Asian countries. China, Indonesia, and India are the three countries with the highest consumption of noodles across the world. Wheat flour is the main ingredient in making noodles. On average, 20–50% of the usage of wheat flour is in the form of noodles (79). In fact, up to 103,620 million servings of instant noodles were consumed worldwide in 2018 (80). Noodles are served as savory dishes globally with the presence of soup or gravy, and instant noodles were also used as space foods (80). Due to the large market of noodles consumption worldwide, researchers have developed noodles with functional properties. Vegetables and fruits by-products have been incorporated into noodles to increase the nutritional value of noodles (Table 3).

**Table 3:** Study and main findings of vegetable and fruit by-products utilization as functional ingredients in development of noodles.

**Product developed**	**Vegetable and fruit by-products**	**Study and main findings**	**References**
Low glycemic index (GI) noodles	Legume seed coat	Legume seed coat (*Bengal gram*) and broken rice were incorporated for the development of low glycemic index noodles. The incorporation of legume seed coat significantly increased the moisture, crude fiber and ash content of noodles. Besides, the total dietary fiber of noodles increased from 5.86 to 9.10% with the incorporation of legume seed coat, in which 2.30% are soluble fiber and 6.80% are insoluble fiber. The incorporation of legume seed coat significantly decreased the glycemic index of noodles, from 66.43 to 56.13.	(81)
Wheat noodles	Pomegranate peel (PGP)	In this study, 0, 0.75, and 1.50% of pomegranate peel (PGP) were incorporated in wheat noodles. The DPPH radical scavenging activity of wheat noodles increased from 23.20% (0% PGP) to 95.16% (1.50% PGP). Besides, the pH of wheat noodles was significantly decreased with the increasing amount of PGP. The color and texture of wheat noodles were altered with the incorporation of PGP, where fortified noodles were darker and harder. Based on the sensory evaluation, no significant difference was shown in terms of firmness and stickiness of all samples.	(82)
Noodles	Apricot kernels (AK)	In the study, 5, 10, 15, 20% of apricot kernel (AK) were incorporated in making noodles. An increased amount of AK being incorporated increased the ash (0.69–1.00%), lipid (0.4–10.6%) and protein (11.5–14.5%). Besides, the color of dried noodles incorporated with up to 15% AK showed no significant difference with control. Nevertheless, the color of raw noodles and cooked noodles were altered with such incorporation. The authors found that the optimum cooking time of noodles decreased with increasing AK amount being incorporated and the total acceptability of noodles decreased with increasing AK addition.	(83)

The incorporation of vegetable and fruit by-products in noodles significantly increased the content of fiber and protein. It is shown that the addition of fruits and vegetables by-products decreased the glycemic index or increased antioxidant activity of noodles as the by-products contained high fiber and phenolic compounds. As the market is lacking a variety of low glycemic food, vegetable and fruit by-products with rich source of dietary fiber could be a useful functional ingredient in exploring more low-glycemic food development.

### Dairy Products

Dairy products such as yogurt, cheese, and butter are produced using milk and are widely consumed across the world. In 2019, 513 million metric tons of milk were produced globally, where the European Union is the highest dairy producer (84). In addition, the European Union has contributed to more than 30% of the global milk production (84). Dairy production is expected to reach 880 million tons in the year 2021 (85). Due to the constantly increasing worldwide trend of dairy products and functional dairy products, more developments of functional dairy products are deemed important.

Vegetable and fruit by-products are well-incorporated into dairy products, such as salad dressing, cheese, and ice cream, and the findings are summarized in Table 4. They are also used as a fat replacer and natural colorant, which showed their wide potential as functional ingredients. As the natural colorant, the incorporation in ice cream showed high acceptability, but more studies could be done on new inventions, which involve a thermal process to indicate the stability of the colorant. In another study, the addition of grape pomace and tomato peels significantly enhanced the antioxidant activities of dairy products, without affecting the organoleptic acceptability of consumers (57, 86).

**Table 4:** Study and main findings of vegetable and fruit by-products utilization as functional ingredients in development of dairy products.

**Product developed**	**Vegetable and fruit by-products**	**Study and main findings**	**References**
Semi-hard and hard cheeses	Grape pomace (GP)	Two formulas were used where 0.8 and 1.6% of GP were incorporated in semi-hard and hard cheeses. No significant changes were observed for the physicochemical parameters of the cheeses after incorporation of GP except the pH value, where it was reduced. The total phenolic content (TPC) and radical scavenging activity (RSA) of cheeses increased with increasing GP content and ripening time. The study showed that semi-hard cheese fortified with 1.6% Chardonnay GP before distillation had the highest TPC and RSA values. In term of microbial counts and proteolysis, no significant difference was observed after GP fortification.	(56)
Yogurt and salad dressing	Grape pomace (GP)	In this study, 1–3% GP, 0.5–1% GP and 1–2% GP were incorporated in yogurt, House Italian salad dressing and Thousand Island salad dressing respectively. During the 3 week storage at 4°C, the fortified yogurt had reduced pH and increased viscosity. Lactic acid percentage and syneresis of fortified yogurt and salad dressing were stable in 3 week storage. In addition, up to 65% peroxide reduction was observed in the fortified products. Besides, the dietary fiber content of fortified samples ranged from 0.94–3.6%, with total phenolic content and DPPH radical scavenging activity respectively ranging from 958–1,340 mg GAE/kg and 710–936 mg AAE/kg. Based on the sensory evaluation, no significant difference was observed in terms of overall liking of all fortified products, compared to the control products. In particular, the fortified Italian dressing showed no significant difference in all attributes including appearance, flavor, texture and consistency.	(57)
Ice cream	Tomato peels (TP)	The carotenoids (lyco-red) extracted from tomato peels (TP) were used as natural colorants and antioxidants for the development of functional ice cream. The study showed that lycopene was the main component of lyco-red extract which contributed to 86.13%, and followed by phytoene, phytofluene and b-carotene. Besides, the degradation of lyco-red increased with the increasing of temperature and decreasing of pH. The authors found no significant difference of lyco-red degradation observed under pH 7 to 10 and temperature ranging from 40 to 70°C. Besides, more than 90 and 50% lyco-red was retained under condition of 100°C (30 min) and pH 2, respectively. 83.80% lyco-red pigments were retained at thermal condition of 100°C for duration of 180 min. The Radical Scavenging Activity (RSA) and Ferric Reducing Antioxidant Power (FRAP) of ice cream increased with the increasing amount of lyco-red. Besides, ice cream supplemented with 5% lyco-red after 30 days storage showed more than 400% higher FRAP value as compared to control. Based on the sensory evaluation, ice cream was tested at storage period of 0 days and 30 days and ice cream with 1 to 4% had score ranged between 87 to 97 which were significantly higher as compared to control with score of 84 at 0 day storage period. Besides, ice cream with 2 and 3% of lyco-red incorporation showed the highest organoleptic acceptability.	(86)
Ice cream	Pomegranate peel (PGP)	In the study, 0.1 and 0.4% of pomegranate peel (PGP) were incorporated in for the development of ice cream. Besides, 2 to 4% of pomegranate seed oil was incorporated as milk fat replacement. It was found that PGP incorporation significantly increased the total acidity, decreased pH and altered the color of ice cream. The use pomegranate seed oil significantly increased the conjugated fatty acid content of ice cream and the incorporation of both PGP and pomegranate seed oil significantly increased the antioxidant and antidiabetic properties of ice creams. Based on the sensory evaluation, increase content of PGP significantly increased score of sour, astringent and color attribute. A significant increase of oxidized flavor was observed with the increasing level of pomegranate seed oil. It was found that ice creams formulation with 0.4% (w/w) PGP and 2.0% (w/w) pomegranate seed oil is suitable for the functional food development.	(87)

### Other Products

As can be seen in Table 5, other types of food have been developed by adding vegetable and fruit by-products, and the addition improved the dietary fiber content. For example, the addition of mango seed kernel (88) and cauliflower by-products (89) improved the nutritional values of traditional Indian cuisines. Other studies have used pomace (26, 28) to improve the product. For the development of jam (28), tomato pomace addition increased the content of dietary fiber up to 20 times.

**Table 5:** Study and main findings of vegetable and fruit by-products utilization as functional ingredients in development of other products.

**Product developed**	**Vegetable and fruit by-products**	**Study and main findings**	**References**
*Idli* and *mathi*	Mango seed kernel (MSK)	Up to 40% of mango seed kernel (MSK) was incorporated in antioxidant rich *idli* and *mathi*. *Idli* and *mathi* are local Indian snacks which are popular as breakfast food in India. The incorporation of 10% MSK in both *idli* and *mathi* formulation were used for further analysis due to the highest acceptability for all attributes in sensory evaluation as compared to other formulation. It was found that 10% of MSK addition significantly increased the moisture content, crude fiber, crude fat, total ash and energy of both *idli* and *mathi*. The minerals including calcium, iron and magnesium content were significantly increased with 10% MSK incorporation. Besides, the antioxidant activity of *mathi* increased from 31.8% to 37.0% and that of *idli* increased from 31.0% to 35.2% with incorporation of 10% MSK.	(88)
*Dhokla*, pancake and *idli*	Cauliflower by-products	The by-products of cauliflower are used to develop three new functional food recipes. In India, cauliflower is widely consumed or used in various types of cuisine, but the leaves are seldom utilized. The authors developed high fiber food recipes by using cauliflower leaves in making *dhokla*, pancake and *idli*. The dried cauliflower leaves had high fiber and iron content. Besides, the sensory evaluation of the food samples indicated the acceptability toward the incorporation of cauliflower leaves. In particulars, food samples with 2 g of cauliflower leaves flour are more organoleptically accepted.	(89)
Jam	Tomato pomace (TP)	Lyophilized tomato pomace (TP) was incorporated in 4 types of jam formulation. The TP jam without pectin has potential uses in food industry due to its higher stability under high temperature (25–90°C). The TP jam formulations contained total carbohydrate content (17.23–43.81%), proteins (1.32–2.03%), fats (0.09–0.16%) and energy value between 87.1–193.7 kcal/100 g. As compared to commercial apricot jam, the dietary fiber of TP jam was 15 to 20 times higher. Based on the sensory evaluation, TP jam with pectin had higher score in terms of spreadability of jam. TP jams without pectin have comparably higher scores in tomato flavor, tomato odor, sour taste and sweet taste.	(28)
Cake	Apple pomace (AP)	Up to 30% of apple pomace (AP) was incorporated in making cake. It was found that AP contained 10.16mg/g total phenol content and 51.1% total dietary fiber, of which 36.50% was insoluble fiber and 14.60% was soluble fiber. An increased amount of AP incorporation significantly increased water absorption, mixing tolerance index and dough development time while decreased dough stability. Besides, 15% addition of AP in cake formulation significantly increased resistance to extension (336–742 BU) and decreased extensibility values (127–51 mm). The gelatinisation temperature of cake was shown to increase with increasing AP incorporation. In addition, the cold paste viscosity of cake was decreased from 1,760 to 970 BU with addition of 15% AP. Although the incorporation of AP altered the physical quality of cakes, the volume and density of cakes was found to be decreased. Based on the sensory evaluation, no significant difference was found in terms of overall quality with up to 20% AP incorporation. The study found that the incorporation of 25% AP in cake increased the total dietary fiber from 0.47 (control) to 14.20% and total phenol content from 2.07 (control) to 3.15 mg/g.	(26)

## Conclusions: A Way Forward in Utilizing Vegetable and Fruit By-products

Vegetable and fruit by-products are wastes from the agriculture, postharvest, processing, distribution, or consumption stages. Some of the by-products have been used as burning material, constructional material, or animal feed, whereas a large amount is being discarded. There are many types of agriculture by-products from the food industry and have been shown to have high nutritional qualities. By utilizing these by-products as functional food and ingredient, the cost of production can be lowered. Importantly, it can increase food sustainability and is one of the strategies to tackle the arising food security problem. Of great significance, findings from the literature showed that agriculture by-products, including vegetable and fruit by-products can be used to increase and enhance the nutritional value of functional foods. This review illustrates the potential of fruit and vegetable by-product as ingredients of value-added for food interventions. With proper hygiene protocol and processing technologies, utilizing these by-products in the functional food industry will be a good alternative to increase the choices of low-cost functional food in the market for consumers. In addition, more research can be done on the underutilized agriculture by-products to seek more possibilities.

## Author Contributions

KL: writing—original draft. KL, MS, and SS: writing—review and editing. MS and SS: supervision. MS: project administration and resources. All authors reviewed and approved the final manuscript.

## Conflict of Interest

The authors declare that the research was conducted in the absence of any commercial or financial relationships that could be construed as a potential conflict of interest.
